# High-yield synthesis of silver nanowires for transparent conducting PET films

**DOI:** 10.3762/bjnano.12.51

**Published:** 2021-07-01

**Authors:** Gul Naz, Hafsa Asghar, Muhammad Ramzan, Muhammad Arshad, Rashid Ahmed, Muhammad Bilal Tahir, Bakhtiar Ul Haq, Nadeem Baig, Junaid Jalil

**Affiliations:** 1 Institute of Physics, Faculty of Science, The Islamia University of Bahawalpur, Baghdad-ul-Jadid Campus, Bahawalpur 63100, Pakistan; 2 Nanosciences and Nanotechnology Department, National Centre for Physics, Quaid-i-Azam University Islamabad, Pakistan; 3 Centre for High Energy Physics, The University of Punjab, Lahore, Pakistan; 4 Department of Physics, Khawaja Fareed University of Engineering and Information Technology, Rahim Yar Khan, Pakistan; 5 Advanced Functional Materials & Optoelectronics Laboratory (AFMOL), Department of Physics, Faculty of Science, King Khalid University, Abha 9004, Saudi Arabia; 6 Center of Research Excellent in Desalination & Water Treatment, King Fahd University of Petroleum and Minerals, Dhahran 31261, Saudi Arabia

**Keywords:** silver nanowires, high yield, visible luminescence, PET film, transmittance, sheet resistance

## Abstract

Silver nanowires (AgNWs) with ultrahigh purity and high yield were successfully synthesized by employing a modified facile polyol method using PVP as a capping and stabilizing agent. The reaction was carried out at a moderate temperature of 160 °C under mild stirring for about 3 h. The prepared AgNWs exhibited parallel alignment on a large scale and were characterized by UV–vis spectroscopy, scanning electron microscopy (SEM), X-ray diffraction (XRD), and PL spectroscopy. The luminescent AgNWs exhibited red emission, which was accredited to deep holes. The SEM results confirmed the formation of AgNWs of 3.3 to 4.7 µm in length with an average diameter of about 86 nm, that is, the aspect ratio values of the AgNWs exceeded 45. An ink consisting of hydroxyethyl cellulose (HEC) and AgNWs was transferred to polyethylene terephthalate (PET) films by simple mechanical pressing. The PET films retained transparency and flexibility after the ink coating. The maximum transmittance value of as-prepared PET films in the visible region was estimated to be about 92.5% with a sheet resistance value of ca. 20 Ω/sq. This makes the films a potential substitute to commonly used expensive indium tin oxide (ITO) in the field of flexible optoelectronics.

## Introduction

Several optoelectronic devices, such as solar cells, touch screens, LC displays, organic EL panels, light-emitting diodes, and organic light emitting diodes, use transparent electrodes made of sputtered indium tin oxide (ITO) films [1–3]. These films are widely used because of their high transmittance, low sheet resistance, and high electrical conductivity. Yet, they still have some major drawbacks such as high cost, intrinsic brittleness due to the ceramic nature [4], and toxic composition. Moreover, the sputtering process is time-consuming and sputtering make the films brittle, which limits the application in flexible applications [5]. Numerous materials are under consideration to overcome these challenges. In the past few years, certain materials, such as graphene, carbon nanotubes (CNT), conductive polymers, and metallic nanowires, have been tested commercially as alternative to ITO films for flexible optoelectronic devices [6–9]. Amongst them, graphene and carbon materials, particularly CNTs, display low optical transparency and high sheet resistance owing to their greater tube–tube resistance and lower inherent carrier concentration [10].

Today, noble metal nanomaterials are extensively employed owing to their superior conduction properties [11]. Among them only silver nanowires (AgNWs) films outperform ITO films in term of transmittance and electrical conductivity [12]. AgNWs are important as they offer a possibility to overcome light–matter interaction in the visible region. The optical properties of AgNWs are determined by localized surface plasmon resonance (LSPR), which depends on shape, size, and environment of the material [13]. AgNWs have gained much attention in replacing ITO because of their low-cost solution-based fabrication, flexibility, and high optical transparency [14–15]. A large-scale uniform synthesis of AgNWs with high aspect ratio would be greatly preferred because of better plasmonic and optical properties [16]. An effective synthetic method is still needed for high yields of impurity-free AgNWs.

In order to synthesize silver nanowires several methods have been successfully developed, including ultraviolet irradiation, salt-free solution-based, salt-mediated solution-based, photo reduction, hydrothermal, wet-chemical, template reduction (hard and soft templates), and ultrasonic reduction methods [17–22]. Lithographic and hard template methods are used to prepare silver nanowires with well-defined dimensions but both methods produce polycrystalline silver nanowires with rough surface, triggering significant scattering and reducing length propagation, thus affecting the optical properties [23].

The polyol method is considered as the most promising and adaptable method with respect to yield, cost, simplicity, and reaction time for the preparation of AgNWs with higher aspect ratios [24–27]. This method involves the reduction of metallic salts in the presence of a polyol. This synthetic method was first proposed by Figlarz and co-workers for the synthesis of rod-like silver particles. They employed ethylene glycol as polyol to reduce silver salt in the presence of polyvinylpyrrolidone (PVP), which was used a nucleating agent [28]. Silver nanowires with an average diameter of 20 nm and lengths up to 20 µm were synthesized by using a high-pressure polyol method. The transparent film fabricated by these nanowires had a transmittance of 88% and a sheet resistance of 40 Ω/sq, which is a performance below that of ITO films. Hence, there is a need to modify this polyol method to produce extra-large and highly ordered silver nanowires to outperform ITO films. It is important to note that the polyol synthesis yields impurities in the form of silver nanoparticles [29]. These nanoparticles formed as a by-product of the reaction drastically affect the electrical conductivity and transparency of the silver nanowires network, thus limiting the optoelectronic applications [30–31].

Here, a fast one-pot modified polyol protocol [32] was employed to obtain ultrapure silver nanowires. In this facile synthesis, ethylene glycol was used as a reducing agent in the presence of PVP as capping agent. Silver nitrate and CuCl_2_ were used as sources of silver and metallic salt, respectively. The resultant nanowires grew 3.3–4.7 µm in length and 75–97 nm in diameter. A silver nanowire ink was then transferred to PET films whose transmittance was calculated to be up to 92.5%.

## Experimental

### Materials

All required chemical reagents, that is, silver nitrate (AgNO_3_), ethyl glycol, polyvinylpyrrolidone (PVP), copper chloride (CuCl_2_), hydroxyethyl cellulose (HEC), polyethylene terephthalate (PET) film, acetone, and ethanol, were purchased from Sigma-Aldrich. All of them were of analytical grade and were used as purchased without any purification. Ethylene glycol (EG) was used as a solvent in the reactions.

### Preparation of silver nanowires

For synthesizing silver nanowires (AgNWs) with high aspect ratio and controlled diameter, ethylene glycol (EG) was used as a solvent and also acted as a reducer. Silver nitrate (AgNO_3_) was used as source of silver. The stabilizer used in the reaction was PVP, which also acted as a capping agent. In addition to stabilizing and capping, PVP also prevented the agglomeration of silver nanowires. CuCl_2_ was used as a salt precursor providing chloride ions. Chloride ions play a vital role in regulating the growth of AgNWs.

To prepare self-arranged silver nanowires, first, 150 mL of EG was stirred and heated at 160 °C for 1 h in order to remove any residual water from EG. The temperature was controlled by using an oil bath. After 1 h, a trace amount (0.225 mg) of CuCl_2_ was added into the reaction solution at 160 °C. After 15–20 min, 1.936 g of PVP dissolved in 5 mL of EG was added dropwise in the reaction mixture. The addition of PVP lasted for almost half an hour. Eventually, 0.48 g of AgNO_3_ dissolved in 5 mL of EG was added dropwise in the solution over a period of 1 h. Initially, the addition rate of AgNO_3_ in the solution was slow, but at the end the injection rate was increased. The stirring rate significantly affected the synthesis of AgNWs. The whole reaction was carried out under mild stirring.

**Figure 1:**
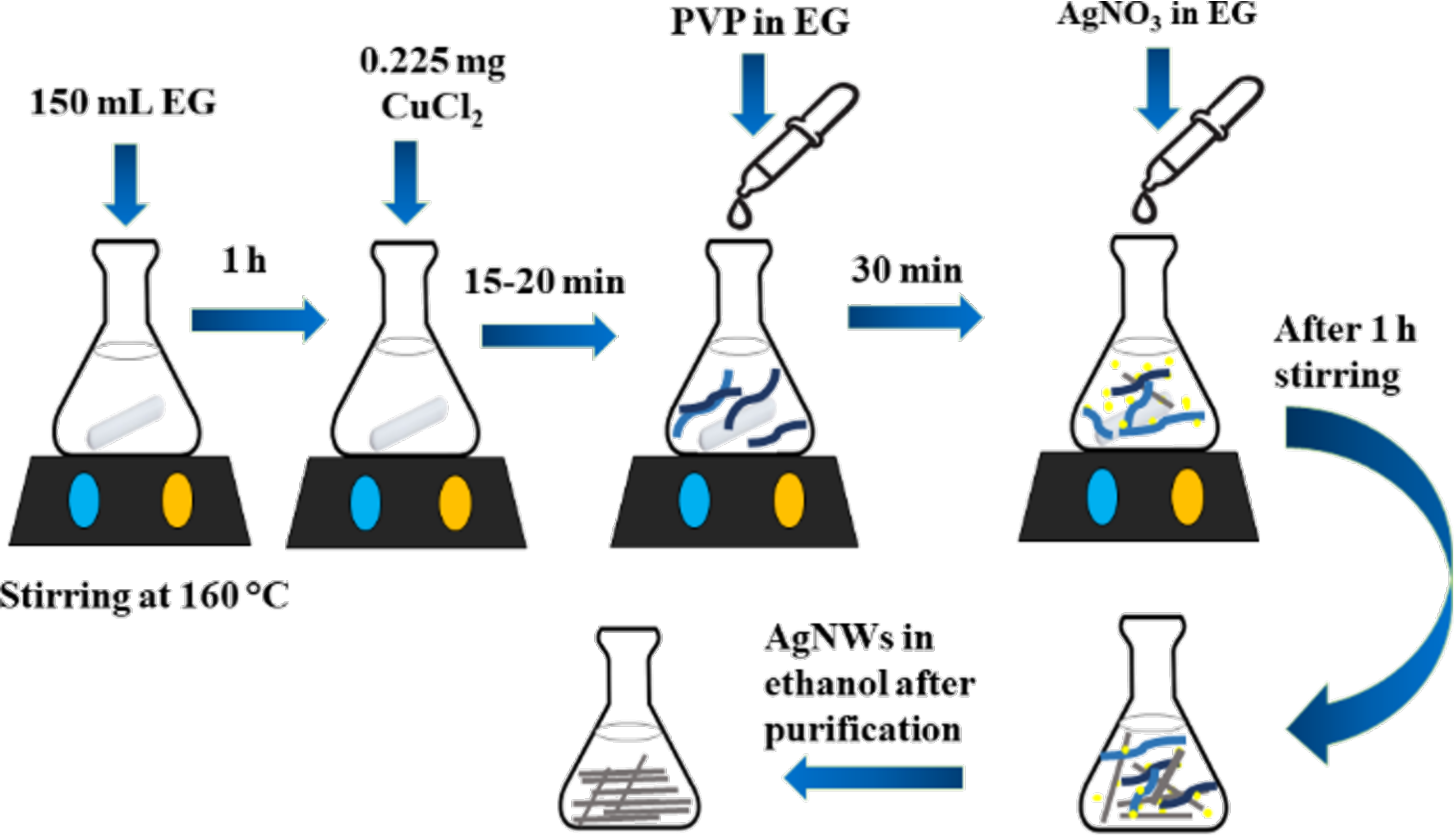
Schematic representation of the modified polyol synthesis of silver nanowires (AgNWs).

The reaction solution undergoes many color changes after the addition of AgNO_3_. Just after the addition of a few drops of AgNO_3_, the solution color changed from transparent to milky gray, then turned to brick red. At the end, the solution color was yellowish white. The yellow color indicated the presence of silver nanoparticles in the solution. The ratio between AgNO_3_ and PVP used in the reaction greatly affects the synthesis of silver nanowires [32]. In the present reaction, the ratio of PVP to AgNO_3_ was 4:1. The solution was then cooled to room temperature by the addition of 30 mL DI water. The cooled mixture was washed through centrifugation twice at 4000 rpm, for 10 min each. After the removal of supernatant, the residual bottom solution showed a silvery shine, which indicated the presence of AgNWs. The final product volume was raised to 0.5 mL by the addition of DI water. The synthesized AgNWs were then used for various characterizations. The prepared solution was further diluted and used for UV–vis spectroscopy and PL spectroscopy. Another batch of the same reaction solution was dried and crushed into fine powder used for SEM imaging. This modified synthesis method is shown schematically in Figure 1 and produces about 270 mg of AgNWs, which could be considered a high-yield synthesis.

### Formulation of AgNW ink and fabrication of transparent conducting films

A facile method was chosen for synthesizing the AgNW ink, which involved the mixing of an aqueous solution of the adhesive agent HEC with AgNWs [33]. First, a 1.0 wt % solution of HEC was prepared in 100 mL of DI water while stirring at room temperature. Then, 15 mL of HEC solution was mixed with 20 mL of AgNW solution in ethanol in a separate glass vial under constant stirring. This was followed by mixing another 15 mL of HEC solution under stirring at room temperature for 2–3 h until the AgNW solution was completely mixed with HEC.

**Figure 2:**
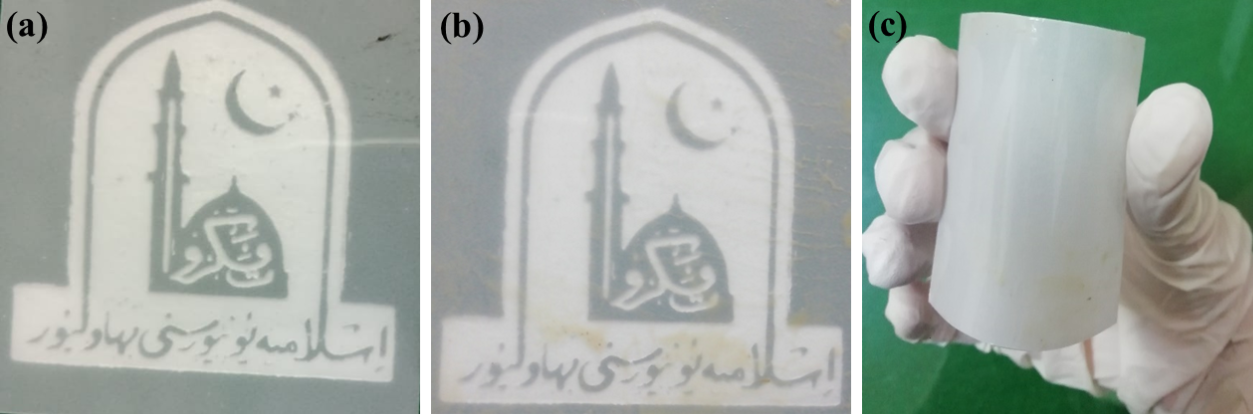
A print of the logo of “The Islamia University of Bahawalpur” behind (a) a PET film without ink coating and (b) a PET film with AgNW ink coating. (c) A photo showing the flexibility of the PET film after AgNW ink coating. These figures clearly depict that PET film retains its flexibility and transparency.

AgNW ink was then transferred to 5 × 5 cm^2^ pieces of PET film by mechanical pressing in order to produce a thin and homogeneous layer of the ink on the surface of the PET substrate. Simply, 0.25 mL of the ink was transferred dropwise on the substrate followed by rolling a stainless steel rod on the PET film for a few minutes. The ink-coated film was then placed in a preheated oven for 3 min for curing. The curing temperature was 130 °C. A higher curing temperature may disrupt the PET film structure and bend the film. The experimental results revealed that after ink coating, the substrate PET film retained its flexibility and transparency, as shown in Figure 2.

### Characterization

UV–vis absorption spectra of liquid AgNW samples were recorded with a Cecil 7500 double-beam UV–vis spectrophotometer in the 400–800 nm wavelength range. A Bruker D8 Advance X-ray diffractometer, operating at 35 mA and 40 keV, was used to scan the sample from 20° to 85° at room temperature. To examine the surface morphology of as-synthesized powder samples, the scanning electron microscopes (Nova Nano SEM 450 operated at an accelerating voltage of 10 kV and ZEISS SEM operated at an accelerating voltage of 20 kV) were utilized. Luminescence measurements were carried out by using a Cary Eclipse MY18060003 photoluminescence spectrometer in the wavelength range of 400–700 nm while exciting the sample at 300 nm wavelength. The transmissivity values of AgNW-loaded PET films were measured using the UV–vis spectrophotometer in visible light wavelength range. A digital multimeter was used to measure the sheet resistance of the PET films. To test the uniformity and electrical conductivity of the PET films, an electrical circuit including a white LED was utilized.

## Results and Discussion

**Figure 3:**
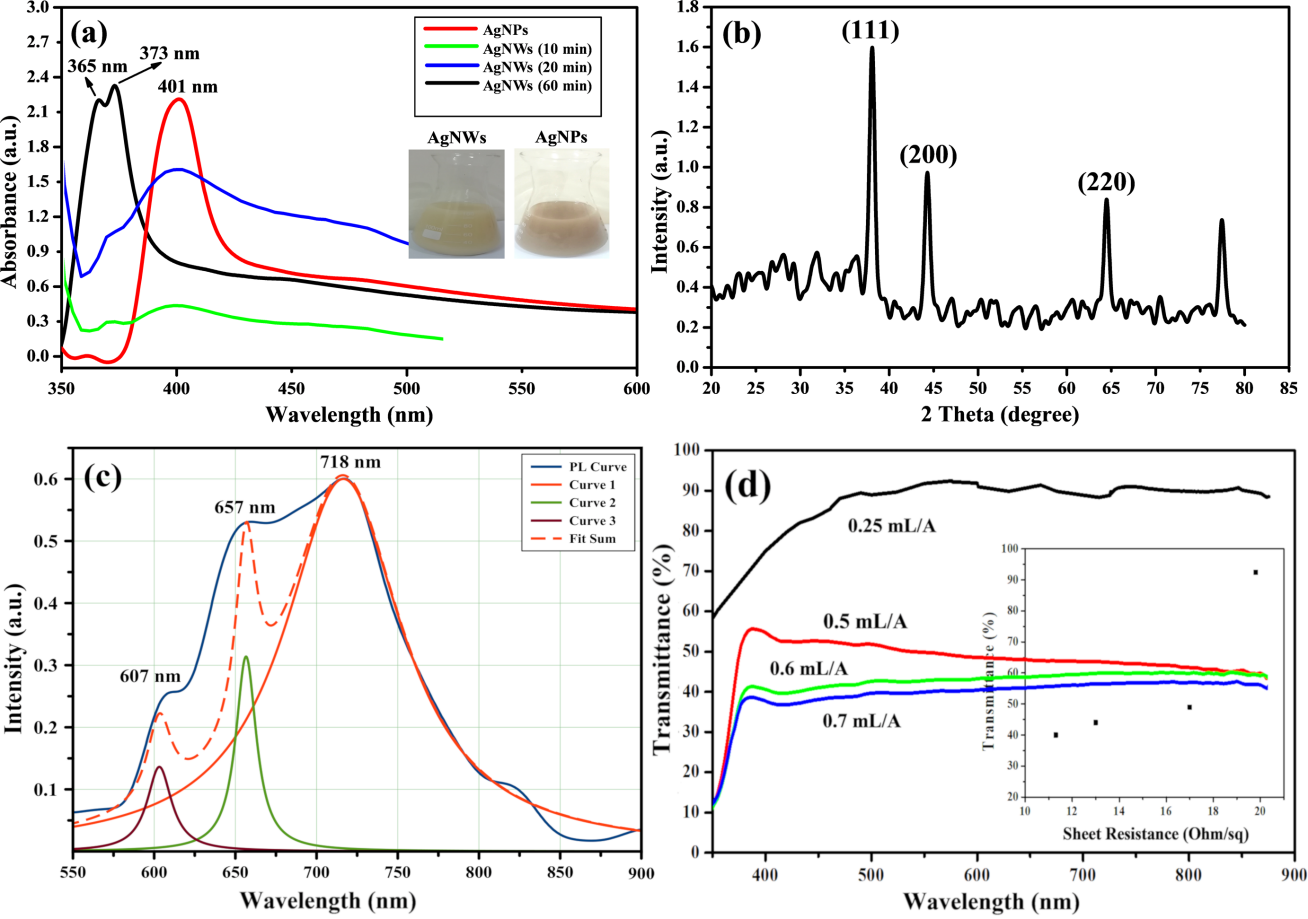
(a) UV–vis absorption spectra indicating the formation of AgNPs just after complete addition of AgNO_3_ (red line) and the end of the reaction with the formation of AgNWs (black line). The spectra after 10 min (green line) and 20 min (blue line) are also given. The insets showing the actual solution colors. (b) XRD pattern of the as-synthesized AgNWs sample. (c) PL emission spectrum of AgNWs falling in the range of 600–800 nm with red emission. The de-convolution into Lorentzian peaks yields peaks at 607, 657, and 718 nm. The red dashed curve is the fit sum of all three Lorentzian peaks. (d) Transmittance-vs-wavelength plot of AgNW-coated PET films with different densities (corresponding to the quantity of AgNW-based dispersion ink, i.e., 0.25, 0.5, 0.6, and 0.7 mL per unit area *A* of the film of 5 × 5 cm^2^) and the inset shows the transmittance-vs-sheet resistance plot of the fabricated PET film samples.

To determine the morphology, the as-prepared silver nanostructures were first characterized by UV–vis absorption spectroscopy. The absorption spectrum of AgNWs is a function of the dielectric material, the chemicals used, and the particle size [34]. Four samples were examined. First, a sample was taken readily after the addition of AgNO_3_ to the reaction solution, and the subsequent samples were taken after 10 min and 20 min, and after washing of the final product (after 60 min of the reaction). Figure 3a shows one sharp peak at 401 nm, which confirms the formation of AgNPs in the solution, while the two SPR peaks below 400 nm are the distinguished feature of AgNWs [35]. As the reaction proceeded to 10 min, the intensity of the absorption peak decreased while a new peak appears at shorter wavelength. This redshift in frequency suggests the growth of rod-like structures, the longitudinal SPR of which might contribute to the advent of a peak at wavelengths shorter than 401 nm. When the reaction approached 20 min, the Ag nanorods further grew in length, and peak intensities continued to rise and become sharp followed by a gradual redshift of the peak positions. An overall hypsochromatic shift of the two plasmonic bands of AgNWs is accompanied by the enhancement of their aspect ratios. The SPR peak at 365 nm may be attributed to the plasmon response along the transverse axis of AgNWs, which is identical to that of bulk silver. The second peak at 373 nm is attributed to the longitudinal plasmon resonance of AgNWs. It is also noted that no other peak was observed, which shows that the final product was free from contamination of any other nanostructures, such as silver nanoparticles or nanocubes. The SEM results also confirm the purity of the product.

The crystalline purity and structure of as-synthesized AgNWs was studied by X-ray diffraction. The different diffraction peaks were matched with the JCPDS card numbers 04-0783 and 99-0094 used for AgNWs. The results clearly indicate that the prepared AgNWs have a face-centered cubic silver crystal structure with the lattice constant *a* = 4.086 Å. The diffraction pattern in Figure 3b shows four distinct peaks with 2θ values of 38.1°, 44.30°, 64.51°, and 77.43°, corresponding to the (111), (200), (220) and (311) Bragg reflections, respectively. No further peaks were observed. This is a clear indication that the final product is free from impurities and no oxidation of silver has occurred. The crystallite size was calculated using the Scherrer equation. The crystal size was found to be 27 nm, which is smaller than the diameter of the silver nanowires calculated from the SEM results.

The photoluminescence spectra of silver nanostructures greatly depend on size and shape of the nanostructures. The PL spectrum in the visible region is associated with deep holes. These deep holes cause green, red, and yellow emissions, whereas shallow holes produce blue and violet emissions. AgNPs have been shown to exhibit green emission at about 540 nm [52]. The PL spectrum of the as-synthesized AgNWs was excited at 300 nm and the spectrum was observed between 600 and 800 nm. In Figure 3c, the PL spectrum of AgNWs shows a broader PL region with high intensity peaks at 657 and 718 nm. These peaks depict transitions at different energy levels within the bandgap. The AgNWs prepared in this experiment give red emission that attributed to deep holes. Figure 3d shows the transmittance spectra of AgNW-loaded PET films. The transmittance (*T*) of the as-fabricated PET films was determined by using the Beer–Lambert equation:

[1] %T=antilog (2−absorbance).

To investigate the effect of the number of coatings on the transparency of the PET film, a range of film samples was prepared with different AgNW ink dispersion volumes (0.25, 0.5, 0.6, and 0.7 mL) and characterized as dispersion volume per unit area (*V*/*A*), where *A* represents the unit area of the film of 5 × 5 cm^2^. We measured the transmittance (*T*) at 576 nm and the sheet resistance (*R*_s_) for all film samples prepared in this work. The sheet resistance (*R*_s_) is commonly defined as the resistivity (ρ) of a sheet of material divided by its thickness (*t*):

[2] $$R_{\text{s}}=\frac{\rho }{t}=\frac{\pi }{\ln2}\cdot \frac{V}{I},$$

where *V* is the measured voltage and *I* is the source current. It was found that for the film with *V*/*A =* 0.25 mL/*A*, *T* approached 92.5% with *R*_s_ approaching 19.8 Ω/sq, in contrast to traditional ITO films with lower electrical conductivity and higher transmittance values. For the film with *V*/*A* = 0.5 mL/*A*, *T* approached 49% with *R*_s_ = 17 Ω/sq. The film with *V*/*A* = 0.6 mL/*A* displayed *T* = 44% with *R_s_* = 13 Ω/sq, and the film with *V*/*A* = 0.7 mL/*A* displayed *T* = 40% with *R*_s_ = 11.3 Ω/sq. These results show that an increase in the number of ink coatings results in a decrease of transmittance and sheet resistance values of the AgNW-coated PET films. A comparison of different parameters of AgNWs synthesized for transparent conducting PET films is presented in Table 1.

**Table 1:** A comparison of different parameters of AgNWs synthesized for transparent conducting PET film to present study.

Synthetic method	Aspect ratio	Transmittance (%)	Sheet resistance (Ω/sq)	Ref.

one-step synthesis	2500	88.20	3.5	[33]
gram-scale Polyol Synthesis	not defined	<90.0	10.0	[36]
wet-chemical synthesis	not defined	85	13	[37]
PVP-mediated polyol process	30–1000	91.5	11.4	[38]
one-pot polyol process	ca. 4000	97.70	155.0	[39]
laser ablation	not defined	90	not defined	[15]
solvothermal method	250–400	89.9	58.0	[40]
polyol synthesis	2000	99.1	130.0	[29]
one-pot polyol synthesis	1009.2	81.6	11.36	[28]
polyol method	900	96.4	24.1	[41]
sonication-induced scission method	ca. 620	93.42	24.1	[42]
dynamic heating method using IR light	not defined	95.0	35.0	[43]
polyol process	625	84.0	15.2	[44]
etching liquid-phase synthesis	ca. 500	93.0	10.0	[45]
polyol process	not defined	86.0	90.0	[46]
coating method	ca. 833	86.0	38.0	[47]
transfer printing and pressing	ca. 375	93.4	11.5	[48]
polyol process	166–583	81.0	130	[49]
bar coating	444–500	90.3	0.745	[50]
Meyer rod method	1000–1250	91.8–93.9	14.4–17.6	[51]
PVP-mediated polyol method	>40	92.5	ca. 20.0	present

**Figure 4:**
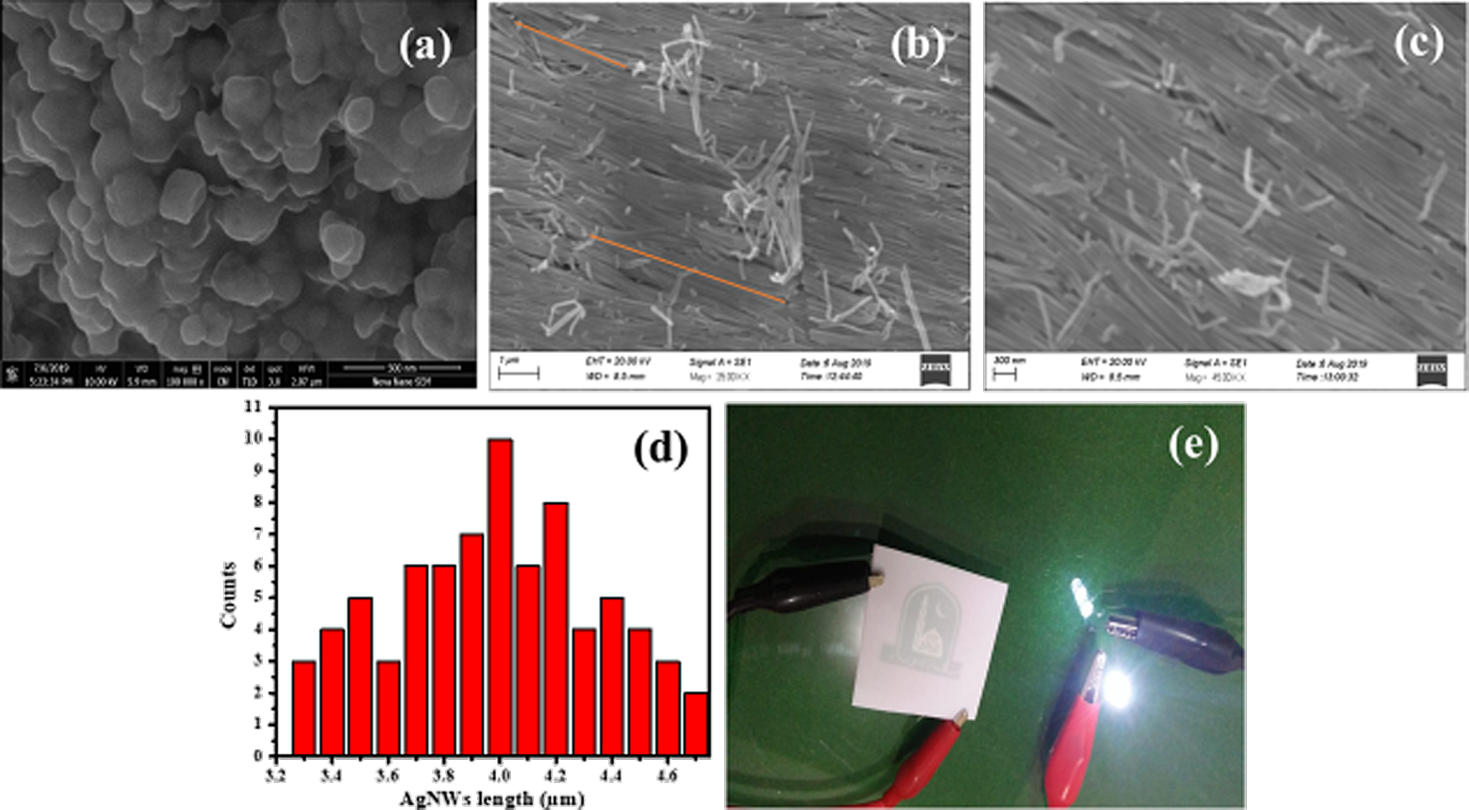
SEM images of (a) AgNPs collected just after complete addition of AgNO_3_ to the reaction solution and (b, c) AgNWs in high yield exhibiting parallel alignment over large regions (collected after 60 min of reaction). (d) Histogram showing the distribution of AgNWs length in the prepared sample. (e) An electric circuit with a lit white LED showing the conductivity of a AgNW-loaded PET film (with sandwiched logo of the institution). The circuit was placed on a glass table with green background.

The SEM analysis of the as-synthesized AgNWs shows that the nanowires exhibit parallel aligned noodle-like structures over large areas (Figure 4b,c). Along with the reaction temperature of 160 °C, the AgNO_3_/PVP ratio has also great effect on the morphology of AgNWs [53]. In our case, a 1:1 ratio of AgNO_3_/PVP started the growth of Ag nanoparticles, as can be seen in the SEM image in Figure 4a. Adding more PVP to the reaction promotes the formation of AgNWs. A AgNO_3_/PVP ratio of 1:4 caused uniform and impurity-free growth of AgNWs. Usually, special attempts are taken to purify the nanowires [39], but in our case no additional steps were required to purify the as-synthesized AgNWs.

The solvent plays a vital role in assembling the AgNWs [54]. It is found that the alignment and density of AgNWs can be easily adjusted by increasing the number of times of water washing. Water, with a very high surface tension of 72.75 × 10^−3^ N·m^−1^ at 20 °C, tends to bundle the hydrophilic AgNWs together [55]. The literature has shown the fabrication of conducting PET films using disordered/unaligned AgNWs. There is a need of fused intersections/connections of nanowires in order to improve the conductivity and transmittance of the PET films [40]. Herein, the end-to-end and side-by-side arranged nanowires with high yield will not require any additional pressing treatments to achieve nanowire interactions. Also, the aligned assembly may not only yield reduced PET film roughness and resistance but also improved transmissivity.

Here, AgNWs with lengths and diameters of 3.3–4.7 µm and 75–97 nm, respectively, have been formed in the reaction. The AgNWs with larger diameters yield lower sheet resistance values if all Ag nanowires in the network are highly connected (at high coverage) [36]. The histogram in Figure 4d shows that some nanowires grow up to 5 µm in length. The aspect ratio calculated turns out to be more than 45. The length of silver nanowires can be controlled predominantly by changing the PVP/AgNO_3_ ratio, as reported earlier [32]. It has been suggested that the parallel arrangement of silver nanowires may improve the conductivity and flexibility of PET films coated with AgNW ink. Thus, more studies are required regarding the alignment of AgNWs on the PET film and the impact on the conductivity and flexibility of the PET film. The flexible and transparent film was bent to demonstrate its curved surface and flexibility (Figure 2c). To test the electrical conductivity of the film, a white LED was used connected to a pair of batteries and the PET film in series to form a closed circuit (Figure 4e).

**Figure 5:**
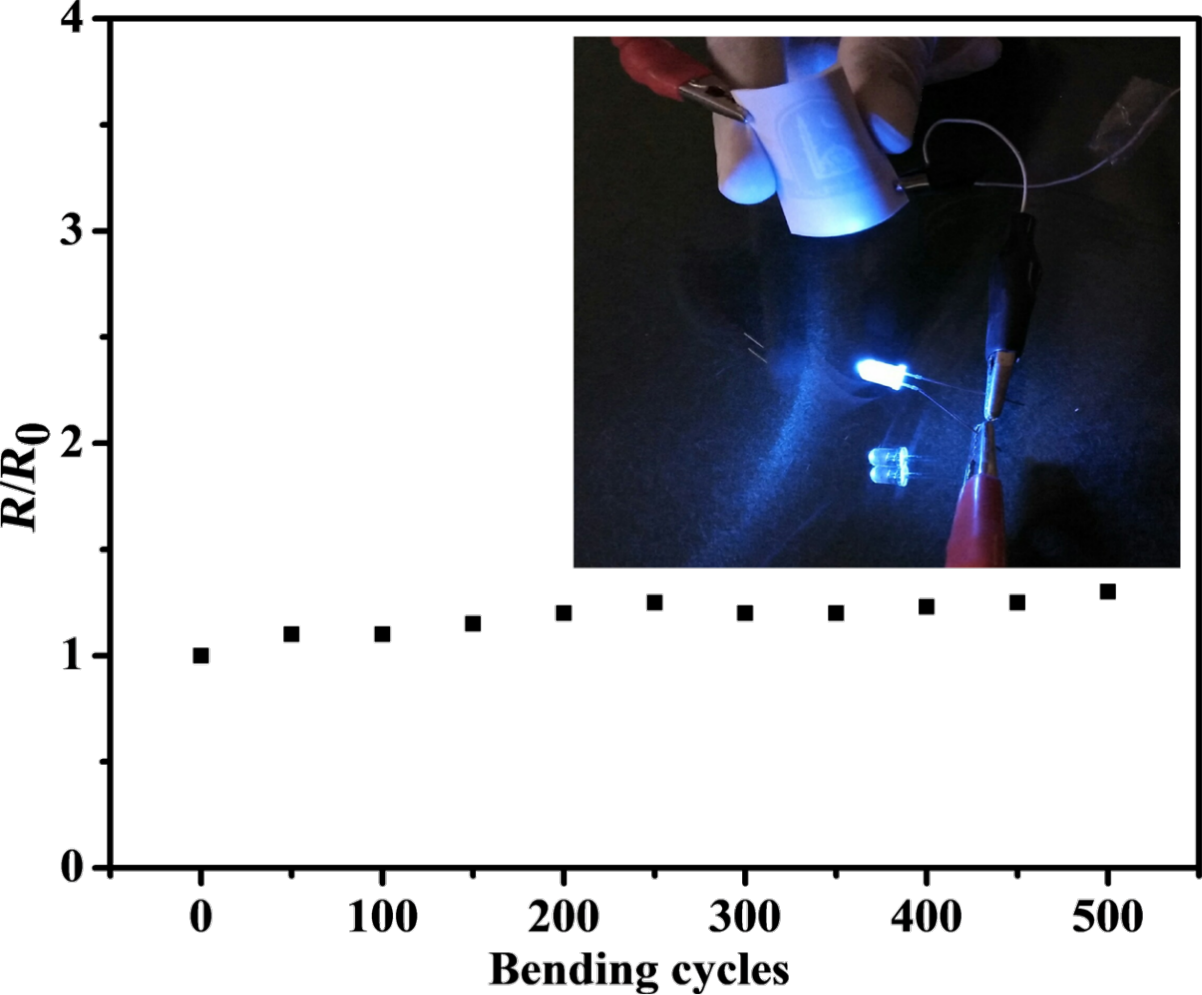
The bending test shows only little change in the sheet resistance of a AgNW-coated PET film after multiple bend–release cycles. The sheet resistance values of the PET film before and after bending test are represented by *R*_0_ and *R*, respectively. The inset shows that the PET film bent by 90° is still conducting.

To demonstrate the mechanical stability of the AgNW-coated PET films, a fabricated PET film was bent repeatedly by 90°. A test measuring the PET sheet resistance during 500 bend–release cycles was carried out. Figure 5 shows that the PET film has almost maintained its original sheet resistance value after 500 bend–release cycles, thus exhibiting a stable electrical performance. The study describes a simple, inexpensive, eco-friendly and non-toxic method to prepare flexible transparent conducting films that could prove very beneficial for flexible transparent optoelectronic devices and could be used in the place of ITO films.

## Conclusion

A high yield of 100% pure and aligned arrays of silver nanowires was obtained by using a convenient template-free polyol method. We prepared AgNWs with an average length of 3.3–4.7 µm and a mean diameter of approximately 86 nm. The morphology of the Ag NWs was best at a reaction temperature of 160 °C and a 1:4 ratio of AgNO_3_/PVP. The XRD analysis confirmed the crystallinity of the AgNWs structures. The aqueous solution of the AgNWs exhibited a broad PL emission band in the red region. The SEM images confirmed that the final product was free from impurities, that is, silver nanoparticles. No other nanostructures could affect the optical and conduction properties, or the roughness of the film. A silver nanowire ink formulated by adding HEC to an aqueous solution of silver nanowires, was then loaded onto the surface of PET films by simple mechanical pressing. The transmittance of the AgNW-coated PET films was calculated to be up to 92.5% at about 20 Ω/sq sheet resistance. This can make it possible to substitute traditional ITO films in flexible optoelectronics technology.
